# Curcumin and Resveratrol: Nutraceuticals with so Much Potential for Pseudoachondroplasia and Other ER-Stress Conditions

**DOI:** 10.3390/biom14020154

**Published:** 2024-01-27

**Authors:** Karen L. Posey

**Affiliations:** Department of Pediatrics, McGovern Medical School, The University of Texas Health Science Center at Houston (UTHealth), Houston, TX 77030, USA; karen.posey@uth.tmc.edu

**Keywords:** nutraceuticals, resveratrol, curcumin, turmeric, dwarfism, growth plate chondrocyte, articular cartilage, joint degeneration, joint pain

## Abstract

Natural products with health benefits, nutraceuticals, have shown considerable promise in many studies; however, this potential has yet to translate into widespread clinical use for any condition. Notably, many drugs currently on the market, including the first analgesic aspirin, are derived from plant extracts, emphasizing the historical significance of natural products in drug development. Curcumin and resveratrol, well-studied nutraceuticals, have excellent safety profiles with relatively mild side effects. Their long history of safe use and the natural origins of numerous drugs contrast with the unfavorable reputation associated with nutraceuticals. This review aims to explore the nutraceutical potential for treating pseudoachondroplasia, a rare dwarfing condition, by relating the mechanisms of action of curcumin and resveratrol to molecular pathology. Specifically, we will examine the curcumin and resveratrol mechanisms of action related to endoplasmic reticulum stress, inflammation, oxidative stress, cartilage health, and pain. Additionally, the barriers to the effective use of nutraceuticals will be discussed. These challenges include poor bioavailability, variations in content and purity that lead to inconsistent results in clinical trials, as well as prevailing perceptions among both the public and medical professionals. Addressing these hurdles is crucial to realizing the full therapeutic potential of nutraceuticals in the context of pseudoachondroplasia and other health conditions that might benefit.

## 1. Introduction

The mechanisms of action of curcumin and resveratrol target many of the pathologic stress mechanisms involved in pseudoachondroplasia (PSACH). Specifically, resveratrol and curcumin, a compound in turmeric, reduce inflammation [1,2,3,4] and oxidative stress [2,5,6,7,8,9,10,11,12,13] and increase autophagy [6,7,14,15]. Testing of a coconut oil dispersion of curcumin, CurQ+ [16], in young MT-COMP mice, a model of PSACH, completely restored limb growth [17]. Resveratrol treatment resolved pain and prevented joint degeneration in adult MT-COMP mice [18]. These findings suggest that resveratrol and curcumin may provide a therapeutic benefit for PSACH. Curcumin/turmeric and resveratrol have a long, safe history of consumption in humans and have been shown to reduce joint degeneration in osteoarthritis (OA) [7,19,20,21,22,23,24,25]. Despite these positive outcomes, there are significant challenges to nutraceutical therapy. In this review, the PSACH pathology will be discussed along with the relevant stresses that curcumin and resveratrol impact [1,10,12,13,14,17,18,26,27,28]. Finally, the hurdles to using natural compounds as therapeutics will be discussed, including one obstacle that is inherent to the compounds: bioavailability. Another barrier is the lack of Food and Drug Administration (FDA) regulation, which leaves the consumer in the dark about the product they are purchasing and creates variation in studies such that they cannot be compared equally. These issues have limited physicians’ interest in using supplements/nutraceuticals and have led to skepticism in the public. Curcumin and resveratrol come from the diet and, therefore, have fewer and less significant side effects than pharmaceuticals and typically cost less than prescription drugs. Overcoming these obstacles could lead to the first treatment for PSACH and perhaps prevention of OA joint damage if caught early enough, safer pain management for joint degeneration, more healthy and active years in late adulthood, and the alleviation of a great deal of the health care burden on society.

## 2. PSACH Pathology

Cartilage oligomeric matrix protein (COMP) is a large, pentameric, matricellular protein that binds to many extracellular (ECM) proteins. COMP contributes to cartilage homeostasis [29,30,31,32] by sponsoring multiple interactions among ECM components, including collagens and proteoglycans [29,30,31]. Mutations in COMP cause PSACH, a severe dwarfing condition characterized by disproportionate short stature (average height of 3′ 9″ (females) and 3′ 11″ (males)) with short limbs [30,33,34,35,36,37,38,39,40,41,42,43]. Joint pain, extreme laxity, and very early-onset joint degeneration are the most significant clinical outcomes [30,33,34,35,36,37,38,39,40,41,42,43].

### 2.1. Chondrocyte Pathology and Mechanisms

**Figure 1:**
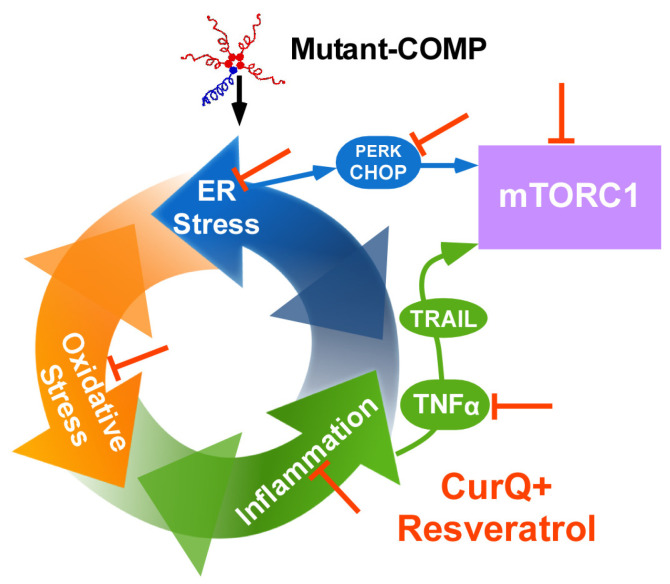
Stress processes involved in mutant-COMP pathology. This schematic briefly summarizes the pathologic processes in PSACH chondrocytes. Mutant-COMP is a pentamer; mutant subunits are shown in red, and wild type is shown in blue. Quality control systems recognize mutant-COMP as improperly folded, and it is held in the ER [47]. This accumulation generates ER stress that leads to oxidative stress and inflammation, and each makes the other stress worse [10]. Prolonged ER stress results in TNFα/TRAIL and CHOP activation that, in turn, elevate mTORC1 signaling, blocking autophagy—thereby preventing the clearance of the ER [11,27]. Orange bar block (⊥) represents inhibition associated with resveratrol and CurQ+, including inflammation, TNFα, oxidative stress, ER stress, PERK, and CHOP [10,12,13,17,18,48].

In order to study PSACH pathologic mechanisms and test therapeutics in vivo, we generated the MT-COMP mouse that expresses mutant human D469del-COMP (deletion of one of five consecutive aspartic acid residues) in tissues expressing type II collagen with doxycycline (DOX) administration. The expression of the most common PSACH mutation (D469del-COMP) in addition to the endogenous wild-type mouse COMP recapitulates the clinical phenotype and PSACH chondrocyte pathology in a mouse designated the MT-COMP [44]. The MT-COMP mouse allowed for us to demonstrate that accumulation of mutant-COMP in the rER (rough endoplasmic reticulum) due to misfolding is cytotoxic to chondrocytes in the growth plate and articular cartilage [38,45,46]. Accumulation of mutant-COMP in chondrocytes induces ER stress, driving both oxidative stress and inflammation, creating a self-perpetuating pathologic loop that leads to an autophagy block [11] (Figure 1). The autophagy blockade is particularly harmful given that autophagy is the primary means to clear the ER of misfolded protein. Autophagy is blocked by high levels of mTORC1 signaling stimulated via TNFα and CHOP [11]. Increased mTORC1 signaling supports general protein synthesis at the detriment of autophagy, directly inhibiting autophagic clearance of the ER in chondrocytes [11,12]. Protein synthesis, in the context of accumulated misfolded protein in the ER, likely exacerbates ER stress [11,12]. In the growth plate, the multitude of stresses driven by mutant-COMP accumulation leads to a loss of chondrocytes needed to generate the matrix necessary to make the cartilage model of long bones, thereby decreasing long bone growth. In articular chondrocytes, the collection of stresses induces degenerative changes in the cartilage related to inflammation, oxidative stress, and autophagy blockade. Moreover, the cooperative action of these stresses drives a senescent phenotype in MT-COMP mice [27]. Senescence is known to propagate degenerative changes to nearby cells and tissues of the joint, hastening joint damage.

### 2.2. PSACH Pain

Pain in the MT-COMP mice has been established through multiple assays, including alterations of gait [27], voluntary running, and grooming [18]. The parents of PSACH young children describe their children as resting more than their peers, tiring easily, and frequently complaining of leg pain that limits stamina (personal communication). This pain in childhood is likely attributable to inflammation-related processes that occur in growth plate chondrocytes due to the accumulated mutant-COMP [34,38,46,49]. PSACH joint pain in adulthood is a chronic problem that affects ambulation, mood, and quality of life [50]. Eighty-one percent of PSACH adults report chronic pain [50]. PSACH pain is described as tiring, exhausting, nagging, aching, throbbing, sharp, and miserable [50]. Joint pain and degenerative changes in the joints along with micro-injuries from extremely lax joints necessitate joint replacements in the late 20′s [36,44]. Typically, hips are replaced first, followed by knees, and some individuals have shoulders and elbows replaced. MT-COMP mice joint degeneration studies demonstrate multiple sources of nociceptive pain present, including subchondral bone remodeling, meniscus damage, synovitis, and inflammation [27]. A non-surgical approach is desperately needed since all joints are affected, but not all joints are replaceable, and numerous years of uncontrolled pain are endured before joint replacement [38,44,50,51].

## 3. Modulation of ER Stress

**Figure 2:**
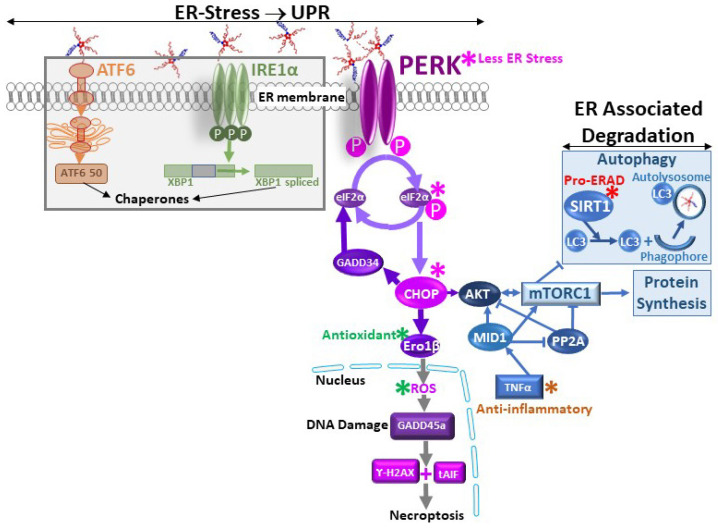
Mechanisms of mitigating ER stress with CurQ+ and resveratrol. ER stress stimulates the unfolded protein response (UPR) that uses three sensors, PERK, ATF6, and IRE1, and only the PERK branch proceeds beyond initial activation with the mutant-COMP response [57] (pink asterisks*). The PERK branch leads to CHOP activation, generating ROS from Ero1β, which is dampened by CurQ+ and resveratrol (green asterisks*) [57]. Of particular importance, the mutant-COMP stimulated increase in PERK, P-eIF2α, and CHOP is reversed by CurQ+ and resveratrol. Less oxidative stress prevents additional ER stress/inflammation driven by ROS. Anti-inflammatory activity of resveratrol and CurQ+ diminishes TNFα [5,12,17,48,58,59,60], lessening mTORC1 activation and exacerbation of oxidative and ER stress (brown asterisks*). SIRT1 upregulation by curcumin and resveratrol [61] promotes ERAD/autophagy, allowing for accumulated protein to be cleared (red asterisks*).

ER stress arises when the capacity of the ER to synthesize correctly folded proteins is exceeded, and secretory cells, such as chondrocytes, are particularly susceptible [52]. In PSACH, the residues deleted or mutated in COMP impact calcium binding, which is crucial for correct folding [53,54,55], leading to a protein that cannot be folded correctly. Three sensors, PERK, ATF6, and IRE1, detect ER stress. BiP selectively binds to misfolded proteins, releasing the sensors and activating downstream targets. PERK suppresses global translation while inducing the transcription of genes involved in the unfolded protein response (UPR), including CHOP, a mediator of apoptosis (Figure 2). Activation of ATF6 and IRE1 triggers the expression of UPR-related targets, such as chaperones and ER-assisted degradation (ERAD). These systems collaborate to restore ER homeostasis. However, if homeostasis is not promptly reestablished, ERAD and autophagy are activated. Cell death or apoptosis is triggered if these mechanisms fail to clear the ER of misfolded proteins.

Mutant-COMP-induced ER stress results in the intracellular retention of mutant-COMP, leading to elevated expression of CHOP and GADD34, consequently reactivating protein translation and exacerbating the intracellular retention of MT-COMP [56] (Figure 2). Reactive oxygen species (ROS) are generated by an increase in endoplasmic reticulum receptor stress-inducible 1 beta (Ero1β) [56]. This oxidative stress triggers DNA damage and upregulates the expression of growth arrest and DNA damage (GADD) genes [56]. The absence of activated caspases coupled with the presence of cleaved apoptosis-inducing factor suggest that mutant-COMP-induced premature chondrocyte death occurs through necroptosis [56] (Figure 2).

Curcumin and resveratrol mitigate ER stress through various mechanisms, including the repression of ER sensors and CHOP expression, the elevation of silent information regulator factor 2-related enzyme 1 (SIRT1), chaperones, and ERAD (Figure 2). Specifically, curcumin and resveratrol decrease the PERK pathway by upregulating SIRT1 expression. This leads to the suppression of activated forms of PERK and eIF2α (Figure 2). Decreasing CHOP indirectly induces autophagy by upregulating SIRT1 by directly stimulating translation of SIRT1 and indirectly increasing SIRT1 levels through phosphorylated AMPKα [61]. This increase in autophagy promotes clearance of mutant-COMP. The activation of SIRT1 by curcumin and resveratrol also inhibits inflammation and supports cellular survival. Furthermore, curcumin and resveratrol enhance heat shock protein levels, aiding in protein refolding [62,63,64]; however, since mutant-COMP cannot be folded properly, this likely does not play a role in this case. ER stress is associated with ROS production and oxidative stress-induced inflammation, and therefore, the antioxidant and anti-inflammatory properties of curcumin and resveratrol indirectly dampen ER stress.

## 4. Anti-Inflammatory Effects

Inflammatory markers are increased in chondrocytes expressing mutant-COMP as early as 2–3 weeks of age [10,27]. Importantly, IL1β, IL6, and TNFα are elevated in MT-COMP mice [10,27], and all of these pro-inflammatory molecules are involved in OA joint degeneration [65,66]. Curcumin exerts its anti-inflammatory effects by reducing various cytokines, including those involved in interleukin (IL) activation, tumor necrosis factor-alpha (TNF-α), and the nuclear factor-kappa B pathway [5,58,67] (Figure 2). On the other hand, resveratrol targets IL-1β, TNF-α, and cyclooxygenase-2 to achieve its anti-inflammatory activity [18,27,48,59,60,68,69,70]. The upregulation of SIRT1 by curcumin and resveratrol plays a pivotal role in driving their anti-inflammatory activities [28,63,71,72,73,74,75] (Figure 1 and Figure 2). In the context of cartilage, TNF-α and IL-1β hold particular significance as pro-inflammatory molecules as they induce the expression of enzymes that contribute to the degradation of extracellular matrix proteins, including matrix metalloproteinases (MMPs) [2,19,20,24,27,48,76,77,78]. The ability of curcumin and resveratrol to modulate these key inflammatory mediators underscores their potential to address inflammation-related issues in cartilage.

## 5. Antioxidant Effects

Oxidative stress has been observed in chondrocytes expressing mutant-COMP [57]. Prolonged ER stress drives the expression of CHOP, which induces endoplasmic reticulum oxidoreductase 1 beta (ERO1β), that works together with protein disulfide isomerase (PDI) to eliminate free radicals [79] (Figure 1 and Figure 2). In vitro oxidative stress in chondrocytes with accumulation of mutant-COMP was demonstrated by increased expression of ERO1β and NADPH oxidase 4 (NOX4) and reduction in mitochondrial membrane potential [57]. Each of these changes indicates that, in the presence of mutant-COMP, there are excessive ROS that drive oxidative stress.

Curcumin and resveratrol play a role in neutralizing free radicals, mitigating the oxidative stress involved in joint damage [10,12,19,28,69,78,80,81,82,83,84,85,86]. Oxidative stress is a key factor in cartilage damage, affecting the tissue at various levels that include damage to cartilage proteins, contributions to chondrocyte dysfunction, and premature cell death. Moreover, oxidative stress induces inflammation that drives painful synovitis, associated with joint degeneration [65,87,88]. Furthermore, inflammation is directly correlated with pain as it stimulates the expression of molecules known to induce pain [89,90]. By addressing oxidative stress and its downstream effects, curcumin and resveratrol may provide a multi-faceted approach to mitigating joint damage, inflammation, and associated pain in conditions like osteoarthritis.

## 6. Cartilage Health

**Figure 3:**
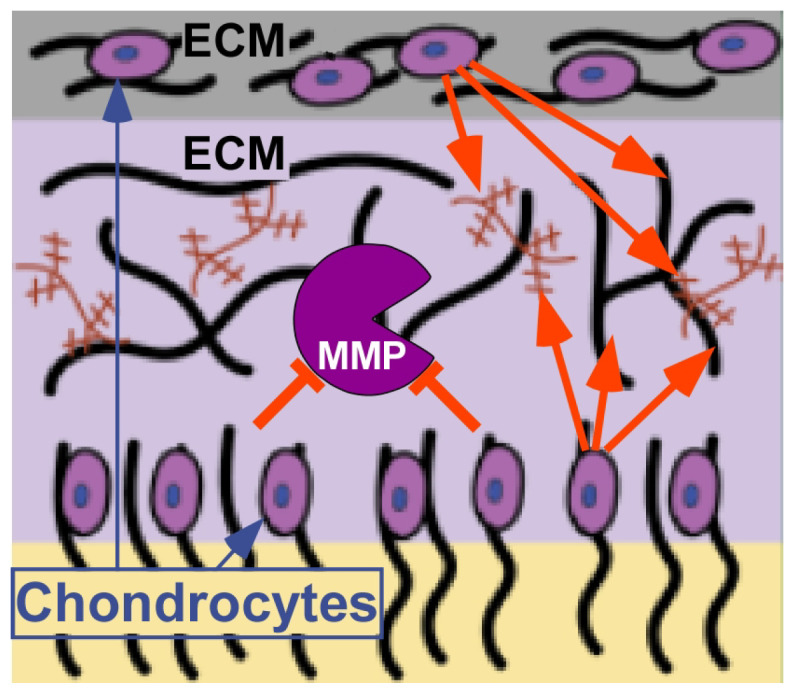
CurQ+ and resveratrol impacts in cartilage. This schematic shows cartilage built of ECM generated by chondrocytes. Orange red block bar (⊥) represents inhibition of MMPs by CurQ+ and resveratrol. Orange arrows depict resveratrol’s enhancement of ECM synthesis. Image shown is modified from https://sitn.hms.harvard.edu/flash/2021/treating-osteoarthritis-the-smart-way/ (accessed on 20 Jan 2004).

MT-COMP mice develop joint degeneration as early as 20 weeks compared to 9–12 months in the background strain, which is similar to the premature joint degeneration observed in PSACH [27]. Numerous studies have demonstrated the chondroprotective effects of curcumin and resveratrol, effectively slowing or inhibiting joint degeneration [1,4,5,6,7,10,12,13,18,19,20,24,27,28,48,77,91,92,93,94,95,96]. Both resveratrol and curcumin enhance SIRT1 and autophagy, mechanisms that safeguard chondrocyte viability—essential for the optimal function of the growth plate and articular cartilage [63,97,98,99]. The deceleration of degradation is likely attributed to reductions in the matrix metalloproteinases (MMP)-1, MMP-3, MMP-13, and ADAMTS5, which are responsible for breaking down collagen and aggrecan, the primary components of cartilage [2,19,20,24,27,48,76,77,78]. Notably, resveratrol has been shown to increase the expression of type II collagen and aggrecan [94,100]. Additionally, both curcumin and resveratrol exhibit the ability to alleviate chondrocyte senescence, mitigate stiffening, and counteract cartilage glycation—factors that contribute to degeneration progression [14,27,48,94]. These multifaceted effects underscore the potential of curcumin and resveratrol as therapeutic agents for mutant-COMP growth plate pathology and the prevention of joint degeneration processes [66] (Figure 1 and Figure 3).

## 7. Pain

MT-COMP mice show signs of pain in multiple assays that are proxies for pain in rodents, including gait changes [27,48], voluntary running, and grooming [18]. Pain modulation can occur at various levels, encompassing the origin of pain, sensory perception, and the transmission of signals. The anti-inflammatory properties of both curcumin and resveratrol play a role in dampening sources of pain [1,3,4,28,87,101,102,103]. Resveratrol specifically inhibits nociceptive (pain-sensing) pathways [104,105,106]. At the same time, curcumin intervenes by inhibiting the activity of key pain mediators, including bradykinin, substance P, and TRPV1 (transient receptor potential vanilloid 1), as well as by modulating the neuronal excitability of sodium channels [20,107,108,109,110,111,112,113]. Moreover, both curcumin and resveratrol alter neurotransmitter levels, influencing the perception and transmission of pain signals to the central nervous system [114,115,116,117,118,119,120,121]. Curcumin modifies serotonin and dopamine levels, whereas resveratrol impacts glutamate and serotonin [116,117,118,119,120,121]. By targeting these multiple facets of pain processing, curcumin and resveratrol present a comprehensive approach to pain modification, potentially offering relief across various dimensions of the pain experience. Importantly, resveratrol treatment of MT-COMP mice abrogates pain (discussed in Section 9) [18].

## 8. CurQ+ Normalizes Limb Growth in MT-COMP Mice

Curcumin, an active ingredient in turmeric, has been used in traditional medicine and cooking for decades. CurQ+, a unique coconut oil-based dispersion technology, is better absorbed, up to 35-fold more, than 95% dry curcumin in capsules [16]. CurQ+ treatment of MT-COMP mice from 1 to 4 weeks postnatally restored IL10 levels, which control MMP13 cartilage degradation and decrease the pro-inflammatory molecules IL6, IL1β, and TNFα [17]. As expected, IL6, IL1β, TNFα, and MMP13, which drive high levels of chondrocyte stress, are all dampened in growth plate chondrocytes with CurQ+ treatment of MT-COMP mice [17]. CurQ+ treatment of MT-COMP mice from 1 to 4 weeks effectively eliminated mutant-COMP accumulation in the ER and ER stress (CHOP), and preserved growth plate chondrocyte viability (TUNEL), autophagy (MID1, pS6), and proliferation (PCNA) [17]. The most important outcome of this study is the normalization of long bone growth in MT-COMP mice with CurQ+ treatment [17]. Restoring long bone growth is the gold standard for treating a dwarfing condition. This dramatic result occurred after three weeks of CurQ+ treatment and is significant given that autophagy blockage jeopardizes chondrocyte viability and proliferation, which are both crucial to growth plate function and long bone growth [17]. Significantly, the dosages of curcumin used in these studies did not negatively impact weight or compromise pup health and are within the range safely consumed by humans.

## 9. Resveratrol Preserves Joint Health and Abrogates Pain in MT-COMP Mice

Previously, we showed that resveratrol treatment of MT-COMP mice from birth to 4 weeks recovers approximately 50% of lost limb growth [10,12]. This partial rescue of limb growth led us to study the effect of resveratrol on premature joint degeneration and pain in MT-COMP mice [18]. Resveratrol was administered to MT-COMP mice beginning at birth, 4, 6, and 8 weeks to 20 weeks. Resveratrol dampens articular chondrocyte stress, as demonstrated by the reduction in ER stress, inflammation, autophagy block, and degenerative enzymes (MMP13) [18]. The inflammatory proteins reduced by resveratrol include TNFα, IL1β, IL6, and IL18 [12,48,60,122]. IL6 and TNFα play a pro-inflammatory role in joint degeneration (OA) [123,124,125], and both IL-6 and TNFα stimulate MMP13 expression [48,76,126], an enzyme that degrades articular cartilage. Clearance of mutant-COMP from the ER of chondrocytes restored homeostasis, normalized function, and alleviated the multiple associated stresses. Early resveratrol treatment (beginning at birth or four weeks) preserved joint health, as joint degeneration scores were equivalent to controls [18]. Specifically, resveratrol treatment decreases synovitis and bone/cartilage damage and diminishes the loss of proteoglycans in the articular cartilage [18]. Importantly, resveratrol treatment ameliorated pain in MT-COMP mice whether administration began at birth or at 4 or 6 weeks [18]. Grooming, a natural behavior, is reduced or less efficient when the mouse is in pain [127]. This assay was validated by the administration of a pain reliever (ibuprofen) or by withholding the induction agent, which normalized grooming scores in the MT-COMP mice [18]. Direct joint pain is associated with synovitis, meniscal damage, and subchondral bone remodeling, while inflammation primarily drives indirect joint pain; all are observed in the MT-COMP mice [58,59]. Given that the MT-COMP mice treated with resveratrol from 6 to 20 and 8 to 20 weeks have joint degeneration in the absence of pain suggests that resveratrol suppresses mutant-COMP-induced pain primarily by dampening inflammation [18]. This study reveals that resveratrol not only improves chondrocyte health but also addresses clinically relevant issues of structural joint degeneration and pain.

## 10. Obstacles to Nutraceutical Treatments

### 10.1. Bioavailability

The low bioavailability of curcumin is primarily linked to its inadequate water solubility, poor absorption, and rapid elimination from the body [128]. CurQ+ has been developed to address these challenges, demonstrating enhanced absorption and elevated serum plasma levels [16,129]. Similar to curcumin, dry resveratrol powder has limited water solubility, a short half-life, and low oral bioavailability [130,131,132]. While liquid resveratrol formulations circumvent the solubility issues associated with desiccation, they introduce complexities by incorporating additional compounds. These co-purifying compounds may be beneficial (or the source of the benefit), but they obscure the specific impact of resveratrol.

### 10.2. Lack of Regulation

Both resveratrol and curcumin are classified as supplements; the United States Federal FDA does not regulate these products. While this may be advantageous to some manufacturers as it eliminates the FDA regulatory burden, the lack of stringent regulations for supplements leads to considerable variations in formulations and concentrations of specific compounds in the product [133]. Our analysis using mass spectroscopy on five different commercially available resveratrol preparations revealed substantial disparities between the reported concentrations and the concentrations measured with mass spectroscopy. The concentrations ranged from 16% to 80% of the labeled concentration. Nature’s Answer liquid resveratrol demonstrated the concentration closest to the reported labeled amount and was selected for use in our resveratrol study [18]. The inconsistency in supplement formulations poses a challenge for consumers in accurately determining the dosage they are actually taking. This lack of uniformity also hinders clinical evaluations of efficacy, as variations in concentrations make it challenging to establish standardized and reliable outcomes.

### 10.3. Perspectives

Prudent skepticism toward any new treatment is justified [134,135]. Communication regarding prescription medications is typically guided by reputable manufacturers and confined to approved uses. The classification of curcumin and resveratrol as supplements constrains manufacturers from making therapeutic or treatment claims. In contrast, naturopathic groups and fringe elements within the medical community have exaggerated claims for supplements, and regulations have not eliminated this problem. Unfortunately, the designation of resveratrol and curcumin as supplements unintentionally fosters a perception among the public that supplements lack the efficacy and potency necessary for legitimate consideration as treatments [136,137]. It also implies that potential dangers do not necessitate stringent regulation, and in the case of resveratrol and curcumin, the risks are low, but other supplements can pose significant risks. Additionally, the low cost of supplements produced by multiple manufacturers and the limited profit potential from a patent introduce financial constraints. The limited profit margins associated with these products hinder the ability to support comprehensive large-scale clinical trials and fund rigorous FDA approval processes.

## 11. Conclusions

Curcumin normalizes growth in young MT-COMP mice [17], while resveratrol prevents early joint degeneration and the associated pain in adult MT-COMP mice [18]. This suggests that these natural products warrant investigation for their efficacy in PSACH. However, conducting clinical trials for a rare condition like PSACH poses significant challenges due to the limited availability of participants. Many individuals with PSACH manage pain using NSAIDs that thin the blood. Complicating matters more, both curcumin and resveratrol possess weak blood-thinning properties, and it is unsafe to combine blood-thinning agents. Consequently, the already restricted pool of participants for a PSACH clinical trial is further diminished.

The multifaceted impacts of curcumin and resveratrol, encompassing anti-inflammatory, antioxidant, pro-autophagy, and chondroprotective effects, pose a challenge in isolating the essential therapeutic attribute for addressing mutant-COMP pathology. Determining whether the improvements in MT-COMP mice are solely linked to a specific therapeutic property or the result from the synergistic cross-talk of these properties presents a complex issue that is impossible to tease out. Our work with the anti-inflammatory aspirin has shown that the stress cross-talk in the mutant-COMP pathology allows for the dampening of one stress to reduce others [10]. While this intricacy of synergistic cross-talk might be perceived as a drawback, especially in the context of FDA applications, it can be argued that the complex pathology of PSACH benefits from a multi-target approach offered by curcumin and resveratrol.

Collectively, these formidable challenges, compounded by the inherent complexities of nutraceutical development, create substantial hurdles for curcumin and resveratrol as potential treatments for PSACH. Despite these impediments, the life-altering impact of intractable pain in PSACH that significantly diminishes the quality of life for affected individuals motivates ongoing research efforts to address this pressing issue.

## Data Availability

Data are available upon request by writing to karen.posey@uth.tmc.edu. K.L.P. is the first and senior author.
